# Self-Myofascial Release Effect With Foam Rolling on Recovery After High-Intensity Interval Training

**DOI:** 10.3389/fphys.2019.01287

**Published:** 2019-10-16

**Authors:** Guillaume Laffaye, Debora Torrinha Da Silva, Arnaud Delafontaine

**Affiliations:** ^1^Complexité, Innovation, Activités Motrices et Sportives, Université Paris-Sud, Université Paris-Saclay, Orsay, France; ^2^Complexité, Innovation, Activités Motrices et Sportives, Université d’Orléans, Orléans, France; ^3^Research Center for Sports Science, South Ural State University, Chelyabinsk, Russia

**Keywords:** foam roller, high-intensity interval training, biomechanical performance, flexibility, delayed onset muscle soreness

## Abstract

The goal of this experiment was to assess the impact of self-myofascial massage with the aid of a foam roller on a lower limb immediately after high-intensity interval training (HIIT), using the Tabata protocol (20 s work/10 s rest, repeated 8 times), according to selected recovery variables. The method used Tabata squats (20 s of air squats/10 s of rest, repeated 8 times), after which the subject performed three series of self-myofascial massage with a foam roller on one leg, the other leg being used as the control. Biomechanical lower limb performance was assessed through a squat jump, a countermovement jump, and a hopping on the spot test. Flexibility was assessed through the active and passive range of motion at the hip, knee, and ankle. Pain was measured by recording the delay of muscle soreness (DOMS). Measurements were recorded immediately after the workout, then 24 and 48 h later. Twenty healthy males participated in the study. The results revealed no effect on jumping performance, in terms of height, leg stiffness, power or force output. Additionally, HIIT had a significant impact on muscle damage, as revealed by the reduction in performance 48 h later (−9.7% for the countermovement height). The self-myofascial release decreased DOMS by 50% for the massaged leg compared with 20% for the control leg and increased the hip range of motion by approximately 4.2% for the massaged leg in comparison with the unmassaged leg. This experiment reveals the poor effect of self-myofascial release on regaining the initial value of performance but could be useful for reducing DOMS after high-intensity interval training.

## Introduction

In recent decades, several studies have investigated foam rolling (FR) as a warm-up or recovery tool (Beardsley and Škarabot, 2015). Foam rollers are used in myofascial release, which includes a wide variety of therapy techniques, including massage and self-massage. Myofascial release is a form of manual soft tissue therapy used to treat somatic dysfunction leading to pain and movement limitation. In self-massage, also referred to as self-myofascial release (SMR), a stick (Mikesky et al., 2002), foam roller (Macdonald et al., 2013), or roller massager (Sullivan et al., 2013) is used in order to practice a massage on one’s own muscles. To illustrate the impact of SMR, studies have focused on the way in which foam rolling affects the range of motion (ROM), muscle soreness, and lower limb biomechanical performance. Thus, the first effect correlates with recovery, whereas the second effect correlates with performance.

Considering the effect of FR on recovery, most investigations reveal that FR has the greatest effect on flexibility, which signifies an increase in ROM after a session of SMR (Sullivan et al., 2013; Halperin et al., 2014; Bradbury-Squires et al., 2015). For instance, Bradbury-Squires et al. (Bradbury-Squires et al., 2015) discovered an increase of 10 and 16% in knee-joint ROM compared with the control group after 20- and 60-s roller-massager sessions on the quadriceps. However, in order to obtain such an effect, a key variable appears to be the way in which the roller was pressed, since the studies that provided no pressure instructions observed lower or insignificant effects compared with studies that took this variable into account (Beardsley and Škarabot, 2015).

The second effect concerning FR which can be found in literature is linked with the performance factor. Most of the cases did not report FR as having any impact on athletic performance during tasks such as vertical jumps, ground reaction force, impulse or rate of force development (Macdonald and Callender, 2011). To date, only one study has found an increase in performance when FR (Peacock et al., 2014) was used as a warm-up combined with a dynamic warm-up as opposed to a dynamic warm-up only, which revealed a low FR impact on the performance. The improvement in recovery is believed to be due to a decrease in soft-tissue stiffness, especially in muscles and in fascia (Mermier et al., 1997; Heyward and Gibson, 2014). Indeed, fascia is composed of connective tissues, primarily collagen, which encloses and separates muscles and other internal organs. It participates in the biomechanics of the musculotendinous system by transmitting force (Benjamin, 2009), and is able to contract. Moreover, fascia contains water: this is expelled when compressed and can therefore affect stiffness. FR has been proposed as a valid tool for reducing stiffness (Schleip and Müller, 2013).

This mechanism appears to have a probable recovery effect through a decrease in the sensation of delayed onset muscle soreness (DOMS) (Macdonald et al., 2014; Pearcey et al., 2015) in trained and untrained athletes, regardless of the tools used or the method used to measure pain. Furthermore, the decrease in the pain sensation after massage is due to the activation of central pain modulatory mechanisms, through neural inhibition mechanisms (Cavanaugh et al., 2017). Indeed, a decrease in contralateral limb pain suggests the contribution of the central pain-modulatory system, which acts to mediate the sensation of perceived pain following brief tissue massage (Aboodarda et al., 2015).

However, little is known about the effect of SMR with the aid of FR on recovery after high-intensity interval training (HIIT), which involves short to long (from 5 to 300 s) intensive work intervals interspersed with active or passive recovery periods (Wiewelhove et al., 2015). This form of training is known to impact cardiomuscular metabolism in a short time. The initial work of Tabata et al. (1996) reveals that 5 d·wk.^−1^ for 6 weeks of HIIT of VO_2max_ with 8 × 20-s sets of exercise at an intensity of 170% with a 10-s rest between each bout increases VO_2max_ by 7 ml·kg^−1^·min^−1^ and anaerobic capacity by 28%. Other research has shown that three weekly sessions of just three 20-s periods of all-out intermittent exercise is sufficient to increase skeletal muscle oxidative capacity and improve cardiometabolic health (Gillen et al., 2014). Furthermore, HIIT induces peripheral fatigue, such as muscle damage, excitation-contraction coupling failure, sarcomere length redistribution, and impaired metabolism, and significantly increases muscle soreness DOMS (Wiewelhove et al., 2015). This process paired with the inflammatory response of muscle fibers after HIIT explains the decrease in performance after a HIIT program, including jump efficiency and the reactive strength index (Pierrynowski, 2007).

Therefore, it seems interesting to assess the impact of myofascial release with FR on recovery after a HIIT program. In fact, to our knowledge (i.e., PubMed and Medline research), the only two studies (Macdonald et al., 2014; Pearcey et al., 2015) that have evaluated the effects of FR after an intense bout of physical activity do not allow us to understand the impact of FR on the recovery parameters. Pearcey et al. (2015) examined the effects of FR on muscle soreness and dynamic performance recovery measures such as sprint speed, agility, broad jump, squat strength, and pain threshold but did not analyze the possible underlying mechanisms. Macdonald et al. (2014) demonstrated that, after exercise-induced muscle damage (back squat maximal performance), FR increased vertical jumps, passive ROM, and muscle activation electromechanical delay, which argues in favor of a beneficial neural response up to 48 h following FR. However, Macdonald et al. (2014) did not evaluate the impact of FR on biomechanical (i.e., leg stiffness, power, and force), psychological (i.e., DOMS, cost of fatigue and cardiac frequency related to participant heart stress) parameters. During hopping on place or running, tendons, muscles and ligaments collectively behave like a linear spring which considers the human body as a mass on a massless spring (Blickhan, 1989), which allows us to characterize whole lower extremity function during stance.

In line with the lack of evidence concerning these parameters, the goal of this study was to assess the effect of SMR with FR after HIIT on recovery according to the following variables: DOMS, range of motion, and biomechanical output during a vertical jump (i.e., power, force, and leg stiffness). We hypothesized that after high-intensity interval training, SMR with FR would: (1) decrease DOMS and thereby (2) increase ROM and biomechanical output.

## Materials and Methods

### Experimental Approach

Twenty healthy men participated in this study. The goal of the experiment was to assess the impact of self-myofascial massage with FR on a lower limb immediately after high-intensity interval training based on selected recovery variables. The method used for HIIT was the Tabata squat protocol (i.e., eight 20-s sets of squats with 10-s rest periods) (Emberts et al., 2013), after which the subjects performed three series of self-myofascial massage with a foam roller on one of their legs, while the other leg served as a control. The term Tabata training is often used synonymously with HIIT (Tabata et al., 1996; Emberts et al., 2013).

**Figure 1:**
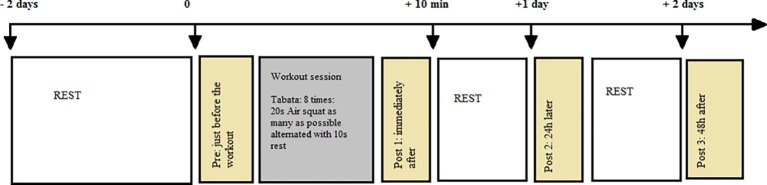
Protocol of the experiment.

Biomechanical lower limb performance was assessed through a one-legged squat jump (SJ) and a one-legged hopping-in-place test (Zemková and Hamar, 2018). Flexibility was assessed through the range of motion at the hip, knee, and ankle. Pain was measured by assessing DOMS. All of these variables were measured at four different time scales: just before the Tabata squats (pre-test measure), immediately afterward (post-test), and then 24 h (post-test 24 h) and 48 h later (post-test 48 h). Resting time (no training) was imposed throughout the protocol (2 days before until the post-test 48 h) as outlined in Figure 1.

### Subjects

Twenty healthy men participated in this study. They were 24.45 ± 3.35 years old, 178.8 ± 9.79 cm tall, weighed 77.42 kilos ±12, and had a fatty mass of 11.97 kilos ±7.3% of their body mass. Recruitment was carried out at a boxing club on the condition that each participant did a minimum of two training sessions per week. The inclusion criteria were: male sex; aged between 21 and 34 years; a regional intermediate boxing level; no boxing training 2 days before the protocol study; no lower limb pain; no medical treatment; and a previous 2-year minimum period of training. The boxing training consists of a 1-h minimum session, including a warm-up, a technical session of heavy-ball and/or speed ball hitting, shadowboxing, rope skipping, and training in the ring with a sparring partner. The non-inclusion criteria were: a recent (in the previous 6 months) traumatic, neurologic, rheumatologic pathology or lower limb surgery which could have interfered with the HIIT performance and the functional tests. The exclusion criteria were: all lower limb injuries that occurred during the protocol study and that could have affected the body region massaged with the foam roller; absence from a protocol session; and non-respect of the protocol and evaluation schedules.

The participants were fully informed about the protocol before participating in this study and signed an informed consent form.

This research protocol was conducted according to the principles of the Declaration of Helsinki on human research and was approved by the ethical committee of Paris-Sud University. Our study was assigned the following trial registration number: 2018-A02892-53.

### Procedures

Each subject observed a 2-day resting period before the test to avoid fatigue effect. The athletes performed 10 min of warm-up exercises starting at low intensity and ending at high intensity; 5 min of running at low intensity (i.e., about 3/10 on the rating of perceived exertion which corresponds to 60–70% of the maximal heart rate); 2 sets of 10 push-ups; 2 sets of 10 air squats; 2 sets of 5 squat jumps; and 2 sets of 5 hopping on the spot jumps, with a 30-s rest between series. The test was performed at the same time each day to avoid circadian variation (Atkinson and Reilly, 1996). The subjects were instructed to perform as many squats as possible under a Tabata protocol: 20 s of air squats with a 10-s rest period, repeated eight times (Tabata et al., 1996).

### Foam Roller Protocol

**Figure 2:**
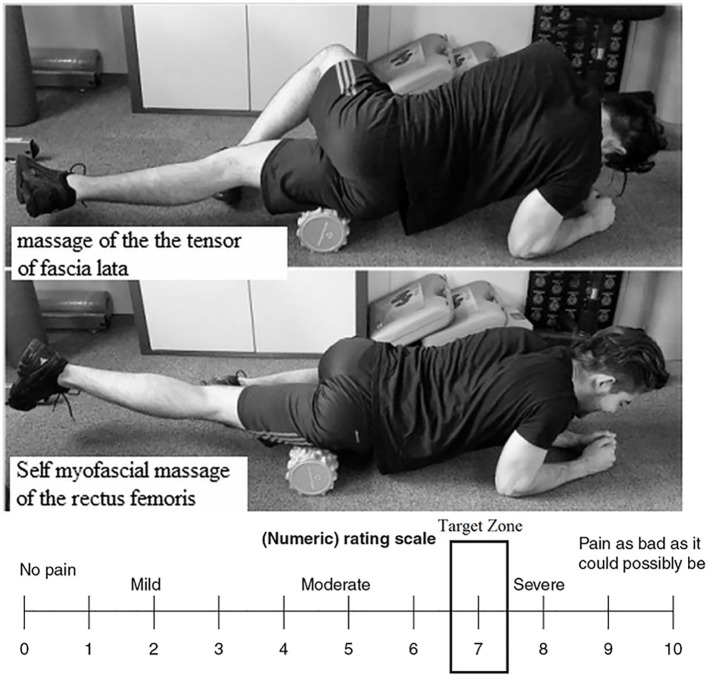
Position of the subject in regard to the two self-myofascial massages: tensor fascia latae and rectus femoris and numerical rating scale target zone (7/10).

We chose a FR similar to that of Macdonald et al. (2014) in order to apply greater pressure on the soft body tissues due to the high-density FR tube (Curran et al., 2008; Macdonald et al., 2013). Immediately after the Tabata squats, and in line with Macdonald et al.’s (2014) FR protocol, each subject performed three sets of SMR using a 16 cm-diameter foam roller, with a picot and thickness of 1 cm. They massaged the tensor fascia latae (lateral side of the leg) from the hip to the knee and the anterior surface of the leg (sartorius and rectus femoris) from the hip to the knee at a velocity of 2 s and an intensity of 7/10 on the visual analogical scale (VAS) (Halperin et al., 2014), in order to ensure that the subjects respected the FR intensity requirement (Figure 2).

The subjects were instructed to stand with their feet a little more than hip-width apart with their toes turned out slightly. They were then asked to bend their knees slowly, pushing their buttocks and hips back and down as if they were going to sit down, while keeping their heads and shoulders aligned with the knees, and knees aligned over the ankles. They then lowered their bodies until their thighs were parallel to the ground (the experimenter verified that the greater trochanter was at horizontal knee level). They were finally instructed to lift their torsos from their thighs and straighten their legs to rise, while lowering their arms back to their sides.

The subjects executed both exercises on the dominant leg for two 60-s bouts each. The dominant leg only was massaged with the FR, while the other leg served as the control. The dominant leg was considered the one on which the subjects always stepped to recover balance following a push from behind (Sadeghi et al., 2000).

### Stress Measurement Outcomes

The heart rate, which represents the participant’s heart stress, is used in order to correlate the heart rate to the VO_2max_ with the aid of the Borg scale, by using two scales: the rating of perceived exertion (RPE) and the Categorical Rating 10 (CR10) (Borg, 1998; Pollatos et al., 2005). RPE scores have seen to be linked to heart rate and VO_2max_ (Eston, 2012) (i.e., HR = 10 × RPE score; Strength = CR10 score × 10). Therefore, the RPE scale was used to assess the exercise intensity. The heart rate was continuously recorded during this test with the use of a polar RS400.

The cost of fatigue during the test was assessed by using the RPE scale for central fatigue (related to the participant heart and lung stress) (Borg et al., 1985) and the categorical rating 10 (CT10) for peripheral fatigue (related to the limb and joint stress) (Pollatos et al., 2005).

### Biomechanical Variables

The ground reaction force, power, jumping performance, and leg stiffness of the lower limbs were assessed on one leg, with the aid of an accelerometric system at a frequency of 500 Hz (Myotest©, Switzerland) in three conditions: (1) squat jump (SJ); (2) countermovement jump (CMJ); and (3) five repeated jumps maximizing jump height and reducing ground contact time to maximize leg stiffness (LS). The participants performed two jumps, keeping their hands on their hips. The performance was calculated by the device on flight time (Choukou et al., 2014). The vertical force and power were assessed by the vertical velocity (Cavagna, 1975), and LS, by the vertical force and displacement (Dalleau et al., 2004). The leg stiffness was calculated as the ratio of maximal force to leg lowering. The leg lowering was calculated by a double integration of the vertical acceleration during the grounding phase. This method has been judged as quite valid (Choukou et al., 2014). The Myotest© was attached to a belt and affixed vertically in the middle of the lower back. The one-leg natural frequency (or preferred frequency) was measured by asking the participants to jump for 10 s (Farley and Gonzalez, 1996). For this purpose, the subjects were instructed to jump on one leg at their preferred frequency, which is considered as the natural oscillation state of the human body.

### Range of Motion

Joint flexibility change was assessed, both actively and passively, at the hip, knee (Macdonald et al., 2013), and ankle in the sagittal plane (flexion/extension) with the use of a universal manual goniometric system (3B scientific, Bartenhiem, France), a validated standard protocol described in literature (Gajdosik and Bohannon, 1987) and often used by clinicians. For the active range of motion, the participants were asked to fully extend their limbs. For the passive range of motion, the practitioner moved the subjects’ limbs and maintained the position, and then asked the participants to relax their muscles before continuing to the end of the movement with the maximal angle.

### Delayed Onset Muscle Soreness

DOMS was measured using the visual analogical scale (Huskisson, 1974), with responses ranging from 0 (no pain) to 10 (maximal pain). Perceived pain during FR was evaluated with the aid of the VAS (Agence Nationale d’Accréditation et d’Evaluation en Santé (ANAES), 2000), while the subjects were performing the FR exercise protocol. The VAS ranged from 0 to 10, with “0” being defined as “absolutely no pain” and “10” being defined as “the worst pain ever felt.” Thirty seconds after the beginning of each 60-s FR trial, the subjects rated their perceived pain for both FR exercises.

### Statistical Analysis

All descriptive statistics were used to verify whether the basic assumption of normality of all studied variables was met. A two-way ANOVA with repeated measures (pre-test, post-test, 24-h post-test and 48-h post-test) was conducted for all of the variables with Eta squared (*η^p^*) to determine the effect size between the massaged leg and the control leg. Fisher’s test *post hoc* comparisons were carried out in the case of significant ANOVA. For statistical analyses, significance was set at *p* < 0.05 and effect size (*η^p^*) was defined as: small for *η*^2^ > 0.01; moderate for *η*^2^ > 0.09; and large for *η*^2^ > 0.25 (Cohen, 1988). All statistics were performed with Statistica 10 software (StatSoft Inc., Tulsa, US).

## Results

### Rating Effect of Perceived Exertion, Categorical Rating 10, and Heart Rate

The HIIT increased both the RPE, from 6 to 16.6 ± 2.25 (*p* < 0.00001), and the value of the categorical rating 10 (CR10), from 0 to 7.8 ± 1.9 (*p* < 0.00001). The heart rate (HR) increased from 77.8 ± 9.7 to 172.8 ± 11.71 (*p* < 0.00001).

### Effect on Squat Jumps

HIIT decreased squat jump height significantly [*F*(3,11) = 40.42; *p* < 0.00001, *η*^2^ = 0.51] from 18.2 cm just before the test to 15.6 cm, whereas neither a massage effect [*F*(1,38) < 1] nor an interaction effect [*F*(3,11) < 1] was observed. HIIT decreased power output [*F*(3,11) = 9.84; *p* < 0.00001, *η*^2^ = 0.20] significantly from 23.5 W/kg cm just before the test to 21.22 W/kg, whereas neither a massage effect [*F*(1,38) < 1] nor an interaction effect [*F*(3,11) < 1] was observed. Force output was not affected by either HIIT [*F*(3,11) < 1] or massage [*F*(1,38) < 1] with a mean value of 18.5 N/kg before the test and 18.3 N/kg immediately after the test. A *post hoc* Fisher’s test revealed a significant difference between the pre-test and all of the other conditions (immediately afterward, 24 and 48 h afterward).

### Effect on Countermovement Jumps

The HIIT decreased countermovement jump height [*F*(3,11) = 55.18; *p* < 0.0001, *η*^2^ = 0.46] significantly from 19.75 cm just before the test to 16.97 cm; but neither a massage effect [*F*(1,38) < 1] nor an interaction effect [*F*(3,11) < 1] was observed. The HIIT decreased power output significantly [*F*(3.11) = 10.51; *p* < 0.00001, *η*^2^ = 0.31] from 25.45 W/kg cm just before the test to 22.42 W/kg, whereas neither a massage effect [*F*(1,38) < 1] nor an interaction effect [*F*(3,11) < 1] was observed. The initial value had still not been recovered 48 h afterward (22.97 W/kg, *p* < 0.05]. The force output was not affected by either the HIIT [*F*(3,11) < 1] nor the massage [*F*(1,38) < 1] with a mean value of 18.7 N/kg before the test and 18.5 N/kg just after the test.

### Effect on Leg Stiffness and Natural Frequency

The HIIT significantly decreased [*F*(3,11) = 3.89; *p* < 0.01, *η*^2^ = 0.09] leg stiffness from 26.34 kN/m just before the test to 24.99 kN/m, whereas neither a massage effect [*F*(1,38) < 1] nor an interaction effect [*F*(3,11) < 1] was observed. Curiously, the leg stiffness value increased to 27.64 kN/m 24 h later and to 28.84 kN/m (*p* < 0.05) after 48 h. The natural frequency increased after the HIIT [*F*(3,11) = 3.89; *p* < 0.01, *η*^2^ = 0.09] from 1.98 Hz to 2.12 Hz 24 h afterward and to 2.16 Hz 48 h after (*p* < 0.05). No massage effect was found.

### Effect on Range of Motion

Neither massage nor HIIT changed the ROM for active or passive ankle or knee flexion/extension. However, a HIIT effect was revealed for the hip on active extension ROM [*F*(3,11) = 11.04; *p* < 0.0001, *η*^2^ = 0.22], with a mean value of 15.05° before, 14.22° after, and 16.4° and 18°, 24 and 48 h later, revealing a significant *post hoc* difference between the initial value and the values obtained 24 and 48 h afterward. Similar results were found in active flexion ROM [*F*(3,11) = 9.09; *p* < 0.0001, *η*^2^ = 0.19], with a mean value of 115.8° before, 114.3° after, and 116° and 120° 24 and 48 h later, revealing a significant *post hoc* difference between the initial value and the values obtained 48 h afterward. No massage effect was found.

### Effect on Delayed Onset Muscle Soreness

**Figure 3:**
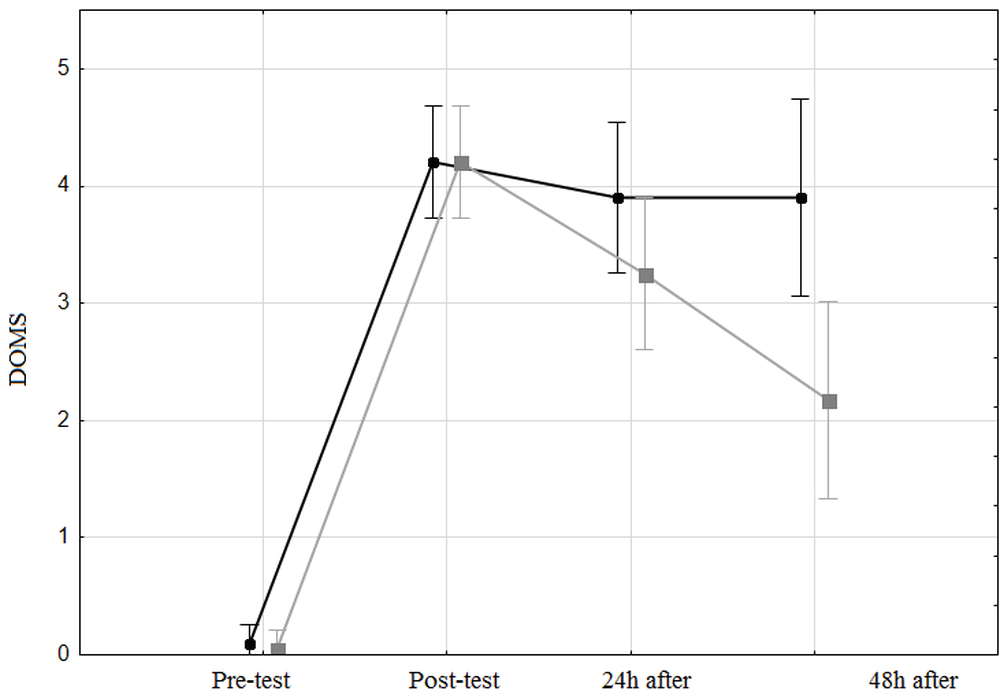
Delay of muscle soreness for both the control leg and the massaged leg, before, just after, 24 h after, and 48 h after the high-intensity interval training. The black line represents the unmassaged leg and the gray line represents the massaged leg.

The HIIT significantly increased [*F*(3,11) = 111; *p* < 0.0001, *η*^2^ = 0.74] the DOMS from 0.07 ± 0.05 just before the test to 4.2 ± 0.16 cm. The FR massage affected the DOMS value [*F*(1,38) = 4.7; *p* < 0.05, *η*^2^ = 0.11] with a mean value of 3.02 ± 0.19 for the control leg vs. 2.42 ± 0.19 for the massaged leg. An interaction effect was observed [*F*(3,11) = 5.4, *p* < 0.002, *η*^2^ = 0.12] between the time and the massaged leg: the DOMS post-test values are similar but decrease after 24 h for the massaged leg contrary to the control leg (Figure 3).

## Discussion

The goal of this study was to assess the role of SMR with FR on the change in DOMS, ROM, and biomechanical variables after HIIT, and the recovery effect of SMR with FR by measuring several variable differences between the massaged leg and the control leg after high-intensity interval training.

**Figure 4:**
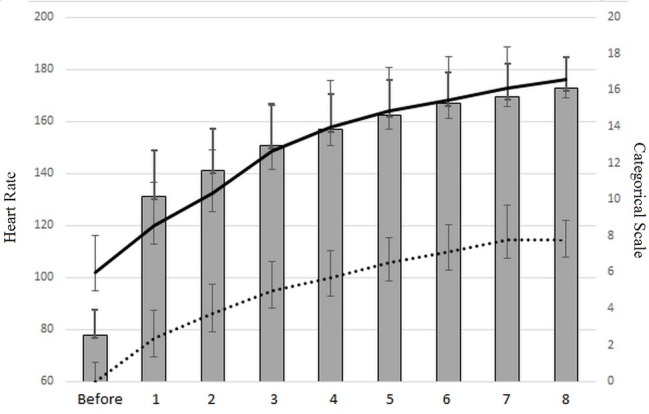
Heart rate evolution (gray histogram), rating values of perceived exhaustion (black curve), and categorical rating score (dotted line).

A correlation coefficient between the heart rate and the categorical rating 10, and between the HR and rating of perceived exertion (RPE) of *r* = 0.90, revealed the well-documented link between the subjective perception of exhaustion and the heart rate (Eston, 2012). Moreover, the high value of both maximal CR10 (*m* = 8.05 ± 1.39) and RPE (*m* = 16.5 ± 2.18) reveals that the HIIT used (Tabata workout) solicits a high level of maximal force, as shown by CR10 and a high level of cardiovascular employment. A value of 8.05 ± 1.39 on CR10 considers the force output to be about 80% of the maximal force, which confirms the high biomechanical constraints of Tabata squats. The mean maximal heart rate value was 173 ± 12 ppm, representing 88% of the theoretical maximum heart rate, calculated with the formula of Gellish et al. (2007) as follows: HR = 207 − 0.7 × age. Figure 4 shows this link between the categorical rate and heart rate during the Tabata workout.

Secondly, SMR with FR had no impact on performance recovery; neither squat jump performance nor the countermovement jump was impacted by massage, with approximately 1% difference between the massaged leg and the control. To date, no study has revealed a positive effect on performance (Macdonald et al., 2013, 2014; Sullivan et al., 2013). A similar ineffective impact on performance has previously been observed on the hamstring by measuring the electrical muscular activity of the muscle after FR intervention (Sullivan et al., 2013). The main explanation for this lack of effect may be related to the fact that SMR only changes the properties of fascia, which contributes to a negligible part of the muscle-tendon unit efficiency, despite the ability of fascia to contract like a smooth muscle (Schleip et al., 2007) due to the presence of myofibroblasts.

Several studies (Macdonald et al., 2013; Sullivan et al., 2013; Halperin et al., 2014) and systematic reviews (Beardsley and Škarabot, 2015; Cheatham et al., 2015) have shown that SMR increases ROM. Similarly, our main result shows that FRM increased hip ROM (i.e., active flexion/extension and passive flexion) by approximately 4.2% for the massaged leg compared with the unmassaged leg. This result is in line with Sullivan et al. (2013) who observed greater hip ROM immediately after two sets of 5 s and two sets of 10 s of SMR. It appears that the hip ROM gain persists over time as shown by the pre- and post-Fisher’s *post hoc* tests carried out 48 h apart. This could be explained by the fact that we also found an increase in the natural jump frequency. These data could be due to the fact that SMR decreased pain in the massaged leg compared with the unmassaged leg as shown in the literature review of Schroeder and Best (Schroeder and Best, 2015).

However, we found no effect of SMR on biomechanical recovery after HIIT. Our subjects lost 10–15% of their biomechanical capacity directly after HIIT and only recovered 3% of it 48 h later. In the present study, this immediate loss of biomechanical capacity and its low recovery after SMR are reflected by the fact that the velocity and power of the CMJ (counter movement jump) decreased in both the SMR and control group after HIIT, and height, velocity, power, and maximal power decreased after the SJ (squat jump) in both the SMR and control group after HIIT.

We can therefore hypothesize that, as a therapeutic tool, SMR is ineffective in increasing motor performance for at least 48 h post-HIIT. To the best of our knowledge, no study to date has assessed the impact of SMR on HIIT. This result contradicts that of Cheatham et al. (2015) who found that SMR increased biomechanical output. We also hypothesized that SMR decreased DOMS after high-intensity interval training. This hypothesis was validated: DOMS decreased by 50% for the massaged leg compared with 20% for the control leg. This positive effect on soreness has previously been documented (Sullivan et al., 2013; Pearcey et al., 2015; Schroeder and Best, 2015) in both trained and untrained samples (Macdonald et al., 2014; Pearcey et al., 2015), using different types of measure, such as pressure pain threshold (Pearcey et al., 2015) and self-reported pain using the BS-11 numerical rating scale (Macdonald et al., 2014). Pain reduction could be due to a central modulation mechanism (Aboodarda et al., 2015; Cavanaugh et al., 2017) or to peripheral adaptation, such as changes in fascia architecture or blood flow (Mori et al., 2004). Mori et al. (2004) have shown that lumbar-muscle self-massage increases muscle blood volume, which stimulates an increase in blood flow in the skin. This results in a rise in temperature, which could accelerate the blood lactate elimination process. Moreover, it has been documented that the lessening of the pain sensation after massage is due to the activation of central pain modulatory mechanisms, through neural inhibition mechanisms (Cavanaugh et al., 2017). A recent study has revealed a decrease in pain in the unmassaged contralateral limb, suggesting the contribution of the central pain-modulatory system, which acts to mediate the sensation of perceived pain (Aboodarda et al., 2015). Our study reveals a statistical difference between the massaged leg and the contralateral one, suggesting a minor role of the central-pain modulatory system. The difference with the aforementioned study is probably due to the high level of solicitation of the cardiovascular and muscular system with the present HIIT, which dramatically increases muscle damage, contrary to a simple intervention on valid muscle. Indeed, the HIIT lowered the biomechanical responses. This result concerning pain could also be explained in literature by another mechanism: SMR appears to modify leg stiffness (Macdonald et al., 2014) through the biomechanical property modification of the leg muscle fascia. The fascia consists of fibrous collagenous tissues that are part of a high-voltage force transmission system (Schleip et al., 2005). The fascia participates in the biomechanics of the musculoskeletal system by transmitting force (Benjamin, 2009), and can contract like a smooth muscle (Schleip et al., 2005). In the fluid flow model, it has been suggested that the water contained in the fascia affects its stiffness, especially when it is expelled during compression. Thus, the compression created by the FR during massage could increase fascia flexibility through temporary changes in the water content, allowing mobilization before the tissue is rehydrated.

However, no leg stiffness modification after SMR was found on either the massaged or unmassaged leg. This result is very surprising since we might expect a positive effect of SMR on leg stiffness due to the increase in natural frequency. Indeed, it is well known that stiffness is closely correlated to jump frequency (Brughelli and Cronin, 2008). The average increase of 7% in natural frequency and of 9% in leg stiffness 48 h after HIIT with no massage effect could be interpreted as a positive metabolic adaptation to the high intensity of the workout. Our results are in line with those of a recent meta-analysis (Wiewelhove et al., 2019), which showed that foam rollers reduced muscle pain perception. However, further investigations are necessary to understand the mechanisms supporting this phenomenon, which could be explained by the following: an increase in plasma endorphins; a decreased arousal level; and an activation of the parasympathetic response and/or placebo effect (Weerapong et al., 2005; Phillips et al., 2018).

Indeed, one limitation in our study is the absence of a placebo group (i.e., sham treatment). The placebo effect could be an alternative hypothesis to explain pain reduction. We could use a “planking exercise” in a placebo group, as Healey et al. (2014). However, this introduces a major bias concerning the aspect of the foam roller sensory effect.

Further study would be necessary with the incorporation of a sham group or a crossover trial (Healey et al., 2014), in which the subjects would pass the foam roller on the skin only (i.e., VAS 0/10), in order to eliminate the sensory effect of the foam roller. However, pressure applied on the skin remains scientifically difficult to measure. Moreover, most athletes are familiar with the use of foam rollers as they are commonly used and publicized (Wiewelhove et al., 2019). This limits the establishment of an efficient placebo to conduct a single- or a double-blind study.

Two other limitations may affect results: the use of an accelerometric system rather than a force plate to measure biomechanical variables during the jump. However, little difference between the two approaches has been demonstrated (Choukou et al., 2014) for the recorded variables.

In conclusion, the main results of this study are that (1) SMR did not impact the recovery of biomechanical variables after high-intensity interval training and (2) SMR decreases the DOMS and increases active and passive range of motion for the hip after HIIT. Consequently, practitioners could use SMR to decrease muscle soreness after HIIT, such as cross-training. This decrease in DOMS could be useful to prevent injury as there are changes in coordination and the level of biomechanical sensibility decreases during workouts with high muscle soreness. Since there is no real consensus on whether stretching after a workout is beneficial or dangerous in this kind of recovery method, SMR could be an efficient alternative to allow athletes to resume training shortly after an intensive workout with severe muscle damage, as previously documented and revealed in our study by the reduction of initial performance 48 h after HIIT. To conclude, trainers could use the increase in active and passive flexibility of the hip to plan workouts, which include movements requiring hip flexibility, such as hurdling, weightlifting, and squatting.

## Ethics Statement

The participants were fully informed about the protocol before participating in this study and signed an informed consent form. This research protocol was conducted according to the principles of the Declaration of Helsinki on human research and was approved by the ethical committee of Paris-Sud University. Our study was assigned the following trial registration number: 2018-A02892-53.

## Author Contributions

GL, DD, and AD designed the study, collected, analyzed, and interpreted the data, drafted and revised the manuscript and figures, and gave final approval.

### Conflict of Interest

The authors declare that the research was conducted in the absence of any commercial or financial relationships that could be construed as a potential conflict of interest.
